# Clonal drift in the aging gut: Mapping the fate of stem cells over time

**DOI:** 10.1371/journal.pbio.3003928

**Published:** 2026-07-23

**Authors:** Tzu-Chiao Lu, Hongjie Li

**Affiliations:** Department of Molecular and Human Genetics, Baylor College of Medicine, Houston, Texas, United States of America

## Abstract

Aging tissue gradually loses its cellular diversity due to stem cell exhaustion. A new study tracks intestinal stem cell lineages across the lifespan revealing that clonal attrition happens during early age in Drosophila.

## Teaser

Aging tissues gradually lose cellular diversity due to stem cell exhaustion. A new *PLOS Biology* study utilizes advanced lineage tracing to track intestinal stem cell lineages across the life span. The findings reveal clonal attrition happens in early ages.

The universal biological truth of aging is a gradual decline in tissue maintenance. In many cases, this is driven by stem cell exhaustion [1]. In the end, it will lead to organ failure or cancer susceptibility. Healthy young tissues feature a diverse, cooperative pool of stem cells. As time ticks on, this competitive landscape narrows: a phenomenon known as “clonal drift”. While this phenomenon has been well characterized in mammalian blood (hematopoiesis) [2,3], tracking the exact and continuous timeline of single-stem-cell lineages in a living organ has traditionally been an experimental black box.

**Fig 1:**
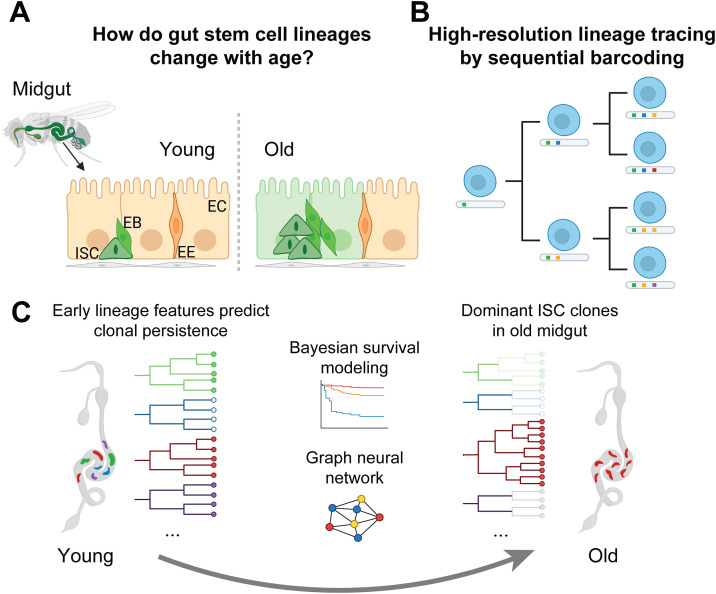
Mapping and predicting stem cell lineage dynamics in the aging fly gut. **(A)** In the *Drosophila* midgut, tissue integrity is maintained by a pool of intestinal stem cells (ISCs). During aging, the gut becomes dysplastic due to ISC overproliferation. **(B)** High-resolution lineage tracing is achieved by recording cumulative somatic mutations onto a genetic cassette. Because daughter cells inherit parent mutations while adding their own, reading these endpoint barcodes allows researchers to infer the branching relationships among sampled cells. **(C)** By applying Bayesian survival modeling and Graph Neural Networks (GNNs), researchers can evaluate clonal persistence and structure evolution. This computational pipeline identifies dominant early-origin lineages that are more likely to persist and expand during aging. Figure created with BioRender.com and modified in Adobe Illustrator.

To tackle this question, researchers require both the right biological system and a highly sophisticated tracking tool. For the system, the *Drosophila* (fruit fly) midgut serves as an ideal model. Structurally and functionally analogous to the mammal small intestine, the fly midgut relies on a dedicated pool of Intestinal Stem Cells (ISCs) to constantly regenerate its epithelial lining. During aging, fly midguts show dysplasia caused by ISC over-proliferation (Fig 1A) [4,5], but how different ISC lineages evolve during aging is largely unknown. Because of the fly’s shorter life span and genetic tractability, it provides an accelerated, real-time window into how stem cells shift during aging.

However, mapping these shifts requires knowing exactly how individual stem cells evolve over several weeks, in living animals. This is very difficult with traditional histological or imaging methods. To solve this, the authors of a new study in *PLOS Biology* leveraged an upgraded version of Substitution Mutation-Aided Lineage Tracing (SMALT), a technology originally introduced in 2021 to map organ development [6]. With this method, Gong and colleagues tracked cell history in order to reconstruct cellular family trees, or phylogenies, of the aging fly gut [7].

How does SMALT do this job? Think of the SMALT system as a black box flight recorder placed inside a cell’s DNA. The authors insert a specific, artificial strip of DNA (3,000 base pairs in the original SMALT; 500 base pairs in the upgraded version) into the organism’s genome. This acts as a “barcode”, and it will record the mutational history via mutations. As the organism grows and cells divide, a specialized system causes substitution mutations to accumulate on this barcode at a very high but controlled rate. Every time a cell divides, it passes its existing mutation down to its daughter cells, which then accumulate new mutations of their own. Then the authors can sequence the DNA and look at their barcodes (Fig 1B). Cells that share the exact same rare mutations are closely related sisters or cousins, while cells with completely different mutations belong to entirely separate branches of the family tree. This study utilizes this technology to look specifically at how these cellular family trees change as the fly gut grows old (Fig 1C).

The authors first used a universal driver (Tubulin-GAL4) to activate lineage recording broadly and examine midgut-wide clonal dynamics from Day 3 to Day 33 after eclosion, the emergence of an adult fly from its pupal case. Across this early adult period, clonal diversity declined progressively. This was supported by multiple diversity metrics, including lineage richness, Shannon diversity, and the reciprocal of the largest clone frequency. These results favor a lineage-extinction model, in which aging is not simply accompanied by proportional expansion of all lineages, but instead by reduced effective contribution from many lineages and increasing dominance of a smaller number of clones.

To focus more directly on stem cells, the authors next used the ISC-specific driver, Dl-GAL4, to label ISCs and their progeny in the posterior midgut from Day 33 to Day 63. This ISC-enriched analysis showed continued loss of clonal diversity during late adulthood. In old flies, only a few major lineages remained detectable. For example, in one old fly sample (Day 63, representing a very old adult fly near the end of its life span, roughly analogous to humans in their 70s–80s), a single ISC lineage represented more than 63% of sampled cells. The lineage trees indicate that fewer ISC lineages continue to contribute effectively to long-term epithelial maintenance.

A particularly interesting finding is that dominant old-age lineages were usually detectable early in adulthood. Most ISC lineages that persisted and expanded in older flies had inferred occurrence times within the first 10 days after eclosion. Bayesian survival modeling estimated the probability that individual lineages would persist to later time points based on growth parameters and tree-structural features. The authors also applied a graph neural network (GNN), specifically an unsupervised graph-convolutional autoencoder, to encode each lineage tree as a numerical representation and identify structurally similar counterparts across successive ages. Together, these computational approaches suggest that lineage topology contains predictive information about future clonal dominance.

This work has several broader implications. Biologically, it shifts attention from late-stage intestinal failure to earlier adult events, when future dominant lineages may already be established. Technologically, it shows how mutation-based lineage tracing can infer temporal stem-cell population dynamics from endpoint samples and generate predictions about long-term clonal fate. Conceptually, it suggests that tissue aging may involve not only changes in stem-cell number or proliferation rate, but also erosion of clonal diversity and effective lineage maintenance.

Several considerations should guide interpretation of these findings. The study was conducted in the *Drosophila* intestine, and the extent to which similar clonal dynamics occur in mammalian tissues remains to be determined. In addition, because different flies were sampled at each age, lineage persistence and GNN-based structural correspondences were inferred statistically at the population level rather than observed by repeatedly following the same clone. Finally, lineage topology alone does not explain why particular clones become dominant, leaving the respective contributions of neutral drift, somatic mutations, epigenetic states, and local niche signals open for future investigation.

By mapping when intestinal clonal diversity begins to collapse, Gong and colleagues [7] provide a temporal framework for stem-cell aging. This study suggests that the aging gut is shaped not only by events in old age, but also by clonal competition established much earlier in adult life.

## Abbreviations

GNN: graph neural network
ISCs: Intestinal Stem Cells
SMALT: Substitution Mutation-Aided Lineage Tracing
